# Immunogenicity and biodistribution of lipid nanoparticle formulated self-amplifying mRNA vaccines against H5 avian influenza

**DOI:** 10.1038/s41541-024-00932-x

**Published:** 2024-08-03

**Authors:** Xiaole Cui, Pieter Vervaeke, Ya Gao, Lisa Opsomer, Qing Sun, Janne Snoeck, Bert Devriendt, Zifu Zhong, Niek N. Sanders

**Affiliations:** 1 Laboratory of Gene Therapy, Faculty of Veterinary Medicine, Ghent University, B-9820 Merelbeke, Belgium; 2 Department of Translational Physiology, Infectiology and Public Health, Ghent University, B-9820 Merelbeke, Belgium; 3 Department of Pharmaceutics, Ghent University, Ghent, Belgium; 4 Cancer Research Institute (CRIG), Ghent University, 9000 Ghent, Belgium

**Keywords:** RNA vaccines, Antibodies, Lymphocyte activation

## Abstract

This study reports on the immunogenicity and biodistribution of H5 hemagglutinin (HA)-based self-amplifying (sa) mRNA vaccines in mice. Four sa-mRNA vaccines encoding either a secreted full-length HA, a secreted HA head domain, a secreted HA stalk domain, or a full-length membrane-anchored HA were investigated. All vaccines elicited an adaptive immune response. However, the full-length HA sa-RNA vaccines demonstrated superior performance compared to head and stalk domain vaccines. The antibody titers positively correlated with the vaccine dose. Cellular immune responses and antigen-specific IgA antibodies in the lungs were also observed. The comparison of the sa-mRNA vaccines encoding the secreted and membrane-anchored full-length HA revealed that anchoring of the HA to the membrane significantly enhanced the antibody and cellular responses. In addition to the injection site, the intramuscularly injected sa-mRNA-LNPs were also detected in the draining lymph nodes, spleen, and to a lesser extent, in the lung, kidney, liver, and heart.

## Introduction

The risk of highly pathogenic avian influenza virus (HPAIV) to both poultry and humans is considerable due to the virus’s extensive circulation, high mortality rates, and potential for transmission between animals and humans. While inactivated virus vaccines are employed in some countries^1–3^ to vaccinate poultry, HPAIV continues to cause outbreaks. In humans, HPAIV can cause severe to deadly infections. Therefore, outbreak preparedness by developing vaccines against avian influenza is recommended. In 2022, HPAIV H5N1 causes more than 131 million domestic poultry lost due to death or culling according to WHO (World Health Organization). H5N1 strains are the dominant HPAIV subtype causing outbreaks in poultry and wild birds worldwide from 2022 October to 2023 September (www.fao.org/animal-health/situation-updates). Nowadays, disease control heavily relies on culling strategies. For poultry the European Union has approved the emergency and preventive use of influenza vaccines, often combined with the “Differentiating Infected from Vaccinated Animal” (DIVA) strategy^4^. The challenge of controlling this disease lies in the rapid mutation rate and antigenic shift, which diminishes vaccine effectiveness. To address this, two conferences are held every year to select new virus vaccine candidates for the upcoming next half year. However, conventional vaccines often suffer from strain mismatches due to the significant delay in response time and the complexity of the vaccine manufacturing process. Therefore, a novel platform that allows a quick and easy antigen modification is urgently needed.

Since 2020, mRNA vaccines have shown remarkable potential and efficiency in preventing the coronavirus disease (COVID-19) pandemic^5,6^. SARS-CoV-2 mRNA vaccines have significantly reshaped the course of the global pandemic, leading to the preservation of a substantial number of lives. The COVID-19 pandemic has ignited a surge in the advancement and utilization of mRNA-based vaccines and therapies. In comparison to inactivated virus vaccines, mRNA vaccines offer several notable advantages over traditional vaccines, including a rapid design, a cell-free manufacturing and the capacity to elicit high antibody levels as well as robust cellular immune responses^7,8^. However, the inherent instability and the size of mRNA necessitate the use of safe and efficient delivery systems to transfer the mRNA in the cytosol of cells. Lipid nanoparticles (LNPs) have emerged as leading non-viral delivery systems for mRNA molecules, playing a crucial role in protecting the mRNA from degradation and delivering the mRNA to the cytosol. Furthermore, LNPs, like synthetic mRNA, possess inherent adjuvant properties that can synergistically enhance the efficacy of mRNA vaccines^9^, thereby augmenting their ability to combat viral infections^10–12^. Self-amplifying mRNA (sa-mRNA) vaccines, typically engineered from an alphavirus vector, encode a viral replicase that temporarily generates new full-length mRNA copies and many copies of a shorter subgenomic mRNA that contains the gene of interest^13^. Sa-mRNAs exhibit superior potential by inducing more potent and sustained mRNA expression in vivo. Consequently, sa-mRNA vaccines can achieve comparable vaccination efficacy to conventional mRNA vaccines while utilizing significantly lower doses^14^. Furthermore, sa-mRNA vaccines hold promise in conferring sufficient protection following a single-dose administration^15^.

Hemagglutinin (HA) is an essential viral protein of influenza virus, characterized by a homotrimer composed of a globular head domain and a stalk domain. As the major glycoprotein on the influenza virion, HA plays a pivotal role in the immune response and serves as a preferred antigen for the development of influenza vaccine platforms^16^, including subunit vaccines, DNA vaccines, and mRNA vaccines^17–19^. Studies have demonstrated that immunization with soluble trimeric HA subunit vaccines induced a potent immune protection against HPAIV H5N1 in mice and chicken through the generation of high levels of neutralizing antibodies^20,21^.

In this study, we demonstrate that LNP formulated sa-mRNA vaccines encoding HA proteins, either in full-length or truncated forms, are capable to induce durable and robust immune response, including high HAI titers, mucosal and Th1-skewed immune responses, in mice, even at a low dose of 0.25 µg. In addition, a comparative analysis of the immunogenicity between sa-mRNA-LNP vaccines encoding cell membrane anchored full-length HA and secreted full-length HA in mice showed superior efficacy of membrane anchored HA. Finally, a biodistribution study of sa-mRNA-LNPs following intramuscular immunization in mice showed high amounts and expression of the sa-mRNA at the injection site, draining lymph nodes and spleen. Our findings highlight the potential of our best-performing sa-mRNA-LNP vaccine as a candidate for combating avian influenza H5 virus infection.

## Results

### Construction and verification of HA encoding sa-mRNAs

**Fig. 1:**
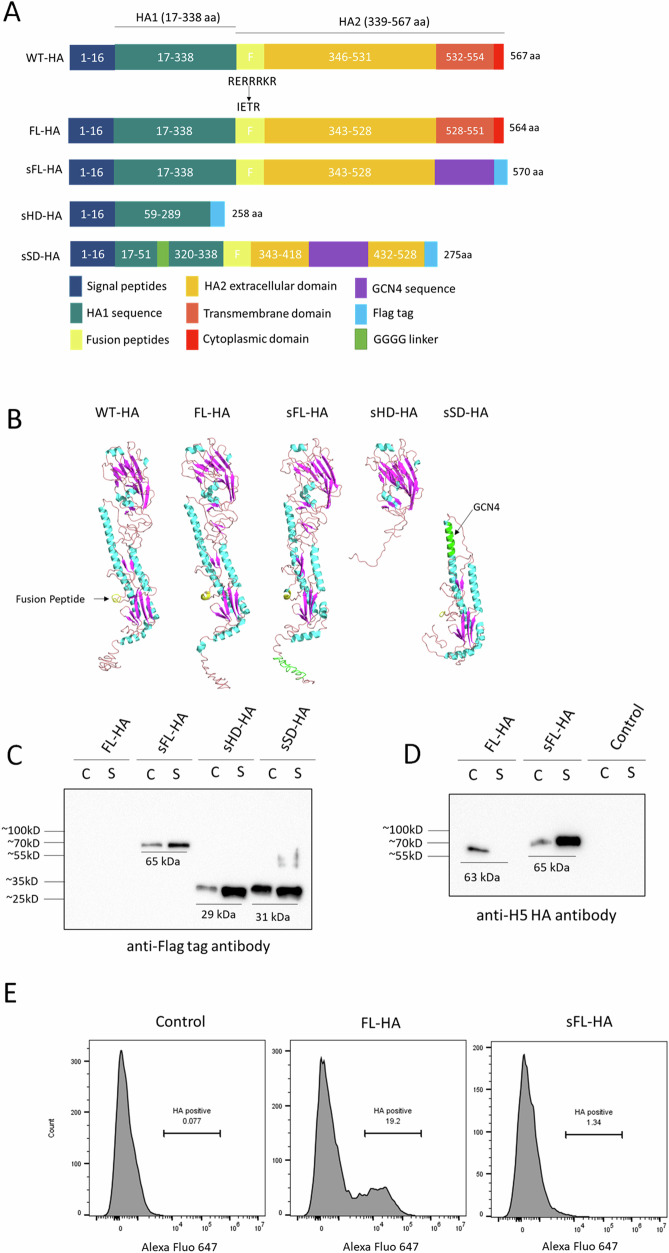
Schematic diagram of the different HA antigens, predicted protein structures, and translation verification of the sa-mRNA vaccines encoding different types of HA using either western blotting or flow cytometry. **A** Schematic overview of the wild type HA and the engineered HA antigens that are encoded by the sa-mRNA vaccines. The numbers within the rectangles indicate the original position of amino acids in the WT-HA sequence. The multi-basic cleavage site (RERRRKR) at the start of the fusion peptide (F) was mutated to IETR in all sa-mRNA vaccine constructs, except sHD-HA. In sFL-HA, the transmembrane domain and cytoplasmic domain were replaced by GCN4 leucine zipper (GCN4), followed by a Flag tag (Tag) sequence (DYKDDDDK). In sHD-HA, amino acids 17–58 and 290–567 were excluded and a Flag tag was added to its C-terminal. In sSD-HA, the amino acids 52–319 of the head domain were replaced by a short linker and amino acids 422–434 were substituted by GCN4. The transmembrane domain and cytoplasmic domain were replaced by a Flag tag. **B** The structures of the HA proteins as predicted by I-TASSER^25^ (available at https://zhanggroup.org/I-TASSER/), in which the fusion peptide is shown in yellow, GCN4 in green, α-helix in cyan and β sheet in purple. **C**, **D** BHK-21 cells were transfected with 1 µg sa-mRNAs encoding different types of HA using Lipofectamine® MessengerMAX™. After 24 h, the HA proteins in cell lysate (c) and supernatant (s) were analyzed by western blot. To detect the HAs, an anti-Flag tag antibody (**C**) or anti-H5 HA antibody (**D**) was used. **E** Flow cytometric detection of surface-exposed HA after transfection of BHK cells with sa-mRNAs encoding luciferase (control), FL-HA or sFL-HA. Surface-exposed HA was visualized 24 h after transfection using an Alexa Fluor-647 conjugated H5N1 HA antibody. The size markers can be found on the uncropped and unprocessed images (Supplementary Fig. S7) used to generate Fig. 1C, D.

A total of four sa-mRNA vaccines were created. Three sa-mRNAs encoded for respectively a secreted full-length HA (sFL-HA), secreted HA head domain (sHD-HA) and secreted stalk domain (sSD-HA). The last sa-mRNA encoded a full-length membrane anchored HA (FL-HA). All vaccines were based on the HA sequence of A/Anhui/1/2005 (H5N1) avian influenza A virus strain (Anhui 05), which belongs to clade 2.3.4. The wild-type HA (WT-HA) has 567 amino acids (aa), consisting of a signal peptide (1–16 aa), HA1 (17–338 aa) and HA2 (339–567 aa) (Fig. 1A). In order to achieve a more stable HA, the highly pathogenic multi-basic cleavage site RERRRKR (338–345 aa) was modified to IETR in all sa-mRNA vaccines except in the sHD-HA^22^. The sFL-HA was constructed by replacing the transmembrane domain and cytoplasmic domain of FL-HA with a leucine zipper GCN4-pII sequence^23^ that can form a trimeric conformation (Fig. 1A). The sHD-HA construct comprised the HA signal peptide followed by the amino acid sequence of WT-HA 59–289 aa. Based on the secreted mini H1 HA #4900, sSD-HA was engineered in which residues 52–319 aa of the head domain were replaced by a four-glycine linker and residues 422–434 aa (based on WT-HA sequence number) were substituted with a GCN4 leucine zipper sequence. Moreover, the transmembrane and cytoplasmic domain were deleted and K323C, S336K, I352T, F405Y, V408I, R410C, F412Y, R418C, T435C mutations were introduced in this sSD-HA^24^. To facilitate detection of the proteins, a Flag-tag was added to the C-terminal of all sa-mRNA constructs, except for FL-HA. Protein structure prediction^25,26^ analysis revealed that FL-HA, sFL-HA, sHD-HA and sSD-HA exhibited similar structures to the WT-HA (Fig. 1B). In vitro transcribed (IVT) and cellulose purified sa-mRNAs (Supplementary Fig. S1A, S1B) encoding FL-HA, sFL-HA, sHD-HA, sSD-HA or luciferase as control were then transfected into BHK cells, and translation of the HA antigens was detected by western blot. As expected, sFL-HA, sHD-HA, sSD-HA proteins with Flag tags were successfully detected in both the cell lysate (C) and the supernatant (S) using an anti-Flag-tag antibody (Fig. 1C). The membrane anchored FL-HA was only detected in the cell lysate (Fig. 1D) with an anti-H5 HA monoclonal antibody as FL-HA was not Flag-tagged. Based on the amino acid sequences the expected sizes of the monomeric forms of the sFL-HA, sHD-HA, sSD-HA and FL-HA antigens, which are respectively, 65 kDa, 29 kDa, 31 kDa and 63 kDa, were in agreement with the molecular weights observed on western blot (Fig. 1C and Supplementary Fig. S1C).

Flow cytometry of BHK cells transfected with sa-mRNA encoding FL-HA, showed binding of Alexa Fluor 647 conjugated anti-H5 HA antibody, whereas no binding was observed on cells transfected with sa-mRNA encoding sFL-HA or luciferase (control) (Fig. 1E). This confirms that the membrane anchored FL-HA protein is exposed at the cell membrane after transfection with FL-HA sa-mRNA.

### Optimization of sa-mRNA-LNPs and dosage for intramuscular injection

**Fig. 2:**
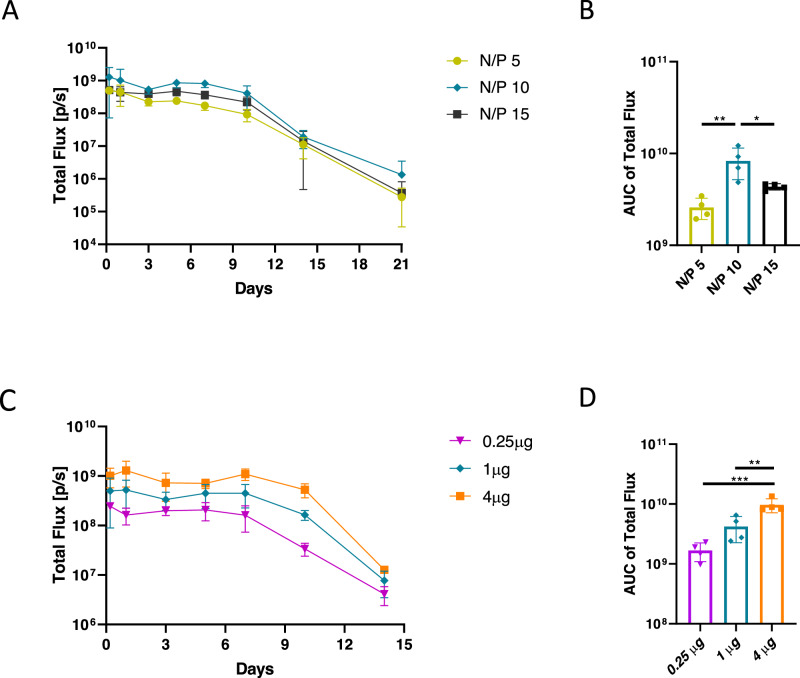
Optimization of sa-mRNA-LNP formulation and dosage. Balb/c mice were injected (IM) with (**A** and **B**) 1 µg luciferase encoding sa-mRNA-LNPs prepared at different N/P ratios (5, 10 or 15), or (**C** and **D**) with different doses of sa-mRNA-LNPs (0.25 µg, 1 µg or 4 µg) prepared at N/P ratio 10. The bioluminescence was monitored with an IVIS imaging system from 6 h to day 21 (**A**, **B**) or to day 14 (**C**, **D**). Total flux (photon/s) reflect the radiance integrated over the area of interest. Bar charts **B** and **D** show the area under the curve (AUC) of the curves in **A** and **C**. Each group contained 4 mice. The significance level (*n* = 4; *p < 0.05; **p < 0.01; ***p < 0.001) was calculated using one-way ANOVA with Tukey’s multiple comparisons test (**B** and **D**). Mean values are shown and error bars represent SD.

We used LNPs containing the same ionizable lipid (ALC-0315) as in the COVID-19 BNT162b2 vaccine to formulate the sa-mRNA vaccines (Supplementary Fig. S2A). In BNT162b2, a smaller non-amplifying mRNA is formulated with LNPs at an N/P (nitrogen of ionizable lipid to phosphate of mRNA) ratio of 6^27^. However, sa-mRNA, which is much longer, may require a different N/P ratio. Therefore, to find the optimal N/P ratio for sa-mRNA, we investigated in mice (*n* = 4) the luciferase production after intramuscular injection of 1 µg sa-mRNA-LNPs encoding luciferase prepared at different N/P ratios (5, 10 and 15). The bioluminescent signal, an indication of luciferase expression, was quantified at different time points using an IVIS system. As shown in Fig. 2A, sa-mRNA-LNP at an N/P ratio of 10 exhibited higher luciferase expression compared to the sa-mRNA-LNPs prepared at N/P 5 or 15 as demonstrated by a significantly higher cumulative luminescence over 21 days (Fig. 2B). Subsequently, a dose-response study using 0.25 µg, 1 µg or 4 µg sa-mRNA-LNPs prepared at N/P ratio 10 showed that the highest dosage of 4 µg significantly outperformed the lower doses (1 µg and 0.25 µg) (Fig. 2C, D).

### Immunogenicity of sa-mRNA vaccines encoding secreted HA antigens

**Fig. 3:**
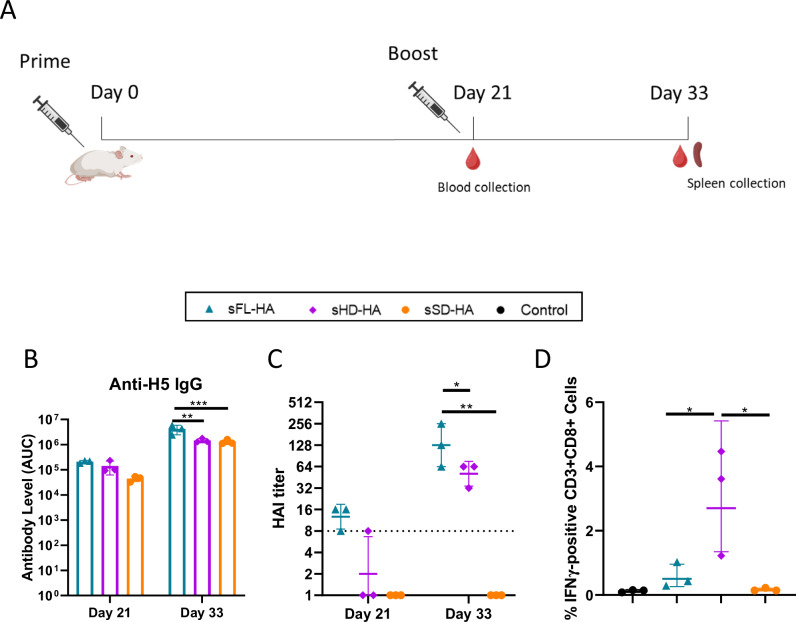
Immunogenicity data of the sa-mRNA vaccines encoding secreted H5 HA antigens. **A** Vaccination schedule. Mice were vaccinated (IM) with 1 µg sa-mRNA-LNPs encoding sFL-HA, sHD-HA, sSD-HA or luciferase (control group) using a prime-boost schedule with a 21-day interval. Blood samples were collected three weeks after the prime (day 21) and twelve days (day 33) after the boost. Spleens were collected twelve days (day 33) after the boost. **B** Serum antibody levels were measured by ELISA and the data are reported as the area under the ELISA absorbance curve (AUC) for each group. No antibody titers were detected in the control group. **C** HAI titers in sera collected three weeks after the prime (day 21) and 12 days (day 33) after the boost. The detection limit is 8 and indicated as a dashed line, and titers below this threshold are considered as undetectable titers and shown as 1. **D** Spleens were harvested 12 days after the boost and stimulated with a peptide pool of a HA from a vaccine-mismatched clade (A/Indonesia/CDC835/2006(H5N1)). Subsequently, the percentage of IFN-γ positive CD3^+^CD8^+^ T cells was assessed by flow cytometry. The significance level (*n* = 3; *p < 0.05; **p < 0.01; ***p < 0.001) was calculated using two-way ANOVA (**B** and **C**) or one-way ANOVA (**D**) with Tukey’s multiple comparisons test. Bars and lines represent the geometric means (**B** and **C**) or arithmetic means. Error bars represent the SD. Figure **A** was created with BioRender.com.

The sa-mRNA vaccines encoding the secreted HA antigens and the control sa-mRNA, encoding luciferase, were formulated in the optimized LNPs (N/P 10) and mice were intramuscularly injected (*n* = 3) using a dose of 1 µg in a prime-boost schedule with an interval of three weeks. Although 4 µg sa-mRNA resulted in the highest expression (Fig. 2C, D), 1 µg was used here to obtain a better discrimination between the vaccines. Antibody levels were measured in the serum three weeks after the prime (day 21) and twelve days after the boost (day 33) (Fig. 3A). After the prime, the highest anti-H5 IgG levels, determined by ELISA and depicted as area under the absorbance curve (AUC), were found in the sFL-HA sa-mRNA vaccine group (AUC, 2.0 × 10^5^). These anti-H5 IgG levels were approximately 1.4 times higher than those in the sHD-HA sa-mRNA vaccine group (AUC, 1.4 × 10^5^) and 4.5 times higher than in the sSD-HA sa-mRNA vaccine group (AUC 4.4 × 10^4^). After the boost, the anti-H5 IgG levels in the sFL-HA group showed a 20-fold increase (AUC, 4.2 × 10^6^) and were significantly higher than the IgG levels in the other groups, which reached AUC values of 1.5 × 10^6^ and 1.3 × 10^6^ in the sHD-HA and sSD-HA group, respectively (Fig. 3B). The sHD-HA group induced a slightly stronger anti-H5 antibody response than sSD-HA group, with over 3 times higher anti-H5 IgG levels three weeks after the prime, but only 1.2 times higher anti-H5 IgG levels twelve days after the boost. Notably, following the boost immunization, the sSD-HA group demonstrated the highest increase in anti-H5 IgG antibody levels, achieving a 29-fold increase compared to the 20-fold and 9-fold increase in the sFL-HA and sHD-HA group, respectively (Fig. 3B). The trends and differences in antibody levels are similar when the results are reported as endpoint titers (Supplementary Fig. S2B). As expected, no detectable anti-H5 antibodies were found in the control group (Supplementary Fig. S2C).

To evaluate the protection capacity of the elicited antibodies, hemagglutination inhibition (HAI) titers were determined using the vaccine-matched Anhui05 H5N1 virus. Mice vaccinated with the sFL-HA sa-mRNA vaccine achieved three weeks after the prime (day 21) a mean HAI titer of 13. Twelve days after the boost (day 33), the mean HAI titer in this group was 150 and significantly higher than the HAI in the other groups. Mice in the sHD-HA group failed to develop a HAI titer above the detection threshold after the prime, but after the boost reasonably high HAI titers that ranged between 32 and 64 were detected. As expected no detectable HAI titers were found in the sSD-HA group (Fig. 3C).

Cellular immunity can play a vital role in conferring cross-strain protection. This prompted us to investigate HA-specific cellular responses. Due to the unavailability of a commercial peptide pool for A/Anhui/01/2005 (H5N1) HA and to demonstrate cross-clade protection of cellular immunity, we chose to employ a peptide pool derived from the vaccine unmatched strain A/Indonesia/CDC835/2006 (H5N1), which belongs to a distinct clade. HA-specific T cell responses were analyzed by intracellular staining of splenocytes isolated on day 12 after the boost vaccination. Mice in the sHD-HA group induced the highest percentage of HA specific T cells, with an average percentage of 3.1% H5 specific IFN-γ positive CD3^+^ CD8^+^ T cells, which is significantly higher than in the sFL-HA group (0.58%). No detectable T cell immunity was observed in the sSD-HA group and control group (Fig. 3D), even not after prolonging the stimulation of the splenocytes with HA peptide pool from 6 h to 48 h (Supplementary Fig. S2D).

### Dose-dependent and mucosal immune responses

**Fig. 4:**
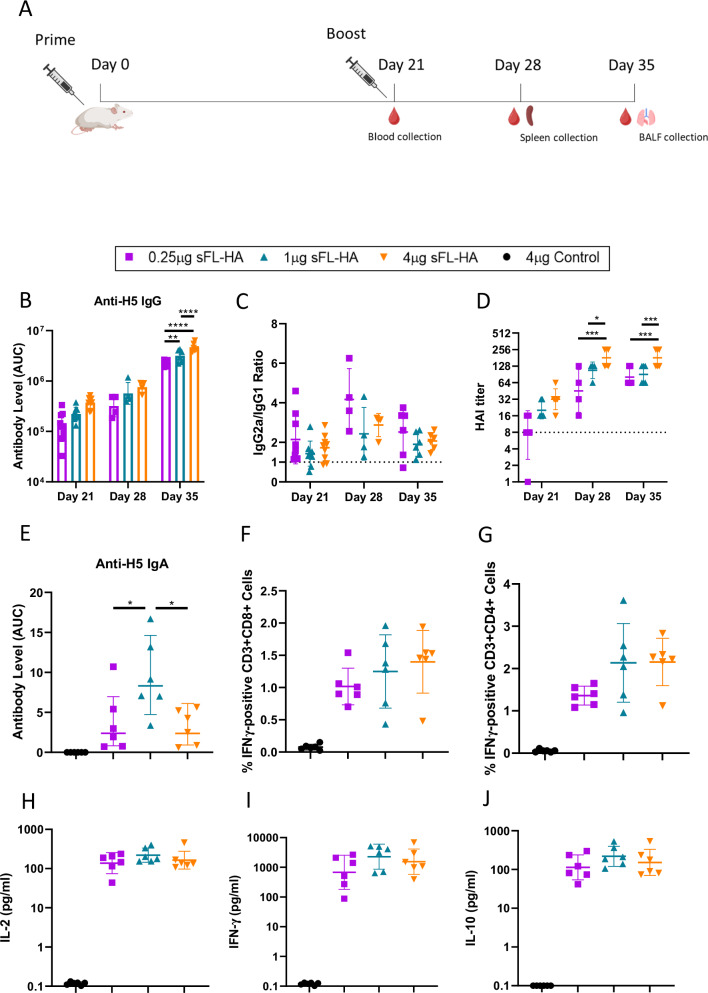
Dose-dependent and mucosal immune responses induced by the sFL-HA sa-mRNA-LNP vaccine. **A** Vaccination schedule. Mice (*n* = 12) were intramuscularly vaccinated with 0.25 µg, 1 µg or 4 µg of sFL-HA sa-mRNA-LNPs using a prime-boost schedule with a 21-day interval. The control mice received 4 µg of a luciferase encoding sa-mRNA-LNP. **B** Serum anti-H5 IgG antibody levels measured three weeks after the prime (day 21 *n* = 9), and one and two weeks after the boost (day 28 *n* = 4 and day35 *n* = 6). Antibody levels are shown as the area under the ELISA absorbance curve (AUC). **C** The IgG2a/IgG1 ratio calculated by dividing the AUC of IgG2a by the AUC of IgG1. Dashed line indicates a value of 1. **D** HAI titers in serum samples taken three weeks after the prime (day 21), and one (day 28) and two weeks (day 35) after the boost. The detection limit is 8 and indicated as dashed line. The value 1 indicated an undetectable titer. No antibodies were detected in the control group. **E** depicts the anti-H5 IgA antibodies in the bronchoalveolar lavage fluid two weeks after the boost. **F** and **G** show the elicited cellular immune responses. Spleens were collected one week after the boost and splenocytes were isolated and stimulated with the HA peptide pool from A/Indonesia/CDC835/2006(H5N1). The percentage of IFN-γ positive CD3^+^CD8^+^ T cells (**F**) and CD3^+^CD4^+^ T cells (**G**) was measured by flow cytometry. The concentrations of IL-2 (**H**), IFN-γ (**I**) and IL-10 (**J**) in the supernatant of stimulated splenocytes was measured by quantitative ELISA. The significance level (*p < 0.05; **p < 0.01: ***p < 0.001; ****p < 0.0001) was calculated using two way ANOVA (**B** and **D**) or one-way ANOVA (**E**–**J**) (day 21 *n* = 9, day 28 *n* = 4, day 35 *n* = 6) with Tukey’s multiple comparisons test. Data are represented as geometric means (**B** and **D**) or arithmetic means. Error bars represent the SD. Figure **A** was created with BioRender.com.

To further investigate a potential correlation between the vaccine dosage and the strength of the immune responses, we immunized mice (*n* = 12) with varying doses (0.25 µg, 1 µg and 4 µg) of the sa-mRNA-LNP vaccine encoding sFL-HA or 4 µg luciferase sa-mRNA-LNP as control, using again a prime-boost schedule with 3 weeks interval (Fig. 4A). Serum antibody (total IgG, IgG1, IgG2a) and HAI titers were determined three weeks after the prime (day 21 *n* = 9), and 7 (day 28 *n* = 4) and 14 (day 35 *n* = 6) days after the boost. This revealed a positive correlation between the dose and both the antibody (total IgG, IgG1, IgG2a) and HAI titers, with the 4 µg group significantly outperforming the 0.25 µg and 1 µg groups on day 28 (Fig. 4D) and day 35 (Fig. 4B and 4D, Supplementary Fig. S3A–K). Moreover, a clear effect of the boost was noticed as two weeks after the booster antibody levels increased more than 10-fold in all groups (Fig. 4B). Notably, the mean HAI titers of the mice that received the 0.25 µg dose increased after the boost to a comparable high and protective mean level of about 85 as obtained with 1 µg sa-mRNA vaccine (Fig. 4D). The ratio of IgG2a/IgG1 consistently remained above 1, suggesting the immune response induced by our sa-mRNA vaccine is skewed towards a Th1 response (Fig. 4C).

Given the aerosol transmission route of influenza, the establishment of robust mucosal immunity is crucial in curbing the viral infection. Therefore, two weeks after the boost (day 35), HA-specific IgA levels in bronchoalveolar lavage fluid (BALF) samples were determined. All vaccinated mice (*n* = 6) had detectable antigen-specific IgA titers in their BALF, with the highest levels found in the 1 µg sa-mRNA-LNP vaccine group, which is significantly higher than other groups (Fig. 4E).

To investigate the effect of the sa-mRNA vaccine dose on the cellular immune responses, spleens were collected 7 days after the booster vaccination, which is 5 days earlier than in Fig. 3D. H5-specific CD8^+^ and CD4^+^ T cell producing IFN-γ were detected in all vaccinated groups after 16 h of peptide pool stimulation, but not in the control group mice. The average percentage of IFN-γ producing CD8^+^ T cells ranged from 1.1% in the 0.25 µg group to 1.46% in the 4 µg group (Fig. 4F). A similar pattern was observed for IFN-γ producing CD4^+^ cells (Fig. 4G), with percentages ranging from 1.33% (0.25 µg) to 2.27% (4 µg). However, these differences in cellular immune response were not statistically significant within vaccinated groups. Antigen-specific T cells that produced IL-4 were not detected (Supplementary Fig. S3L, S3M). Moreover, we also measured multiple cytokines secreted in the supernatant of splenocytes following 16 h of stimulation with the HA peptide pool. Interestingly, the levels of IL-2 (Fig. 4H), IFN-γ (Fig. 4I), and IL-10 (Fig. 4J) increased drastically in all vaccinated mice, and again no clear dose-dependent effect was noticed. In line with the data in Supplementary Fig. S3L, S3M, IL-4 could not be detected in the supernatant of splenocytes stimulated with the HA peptide pool (Supplementary Fig. S3N).

### A head-to-head comparison of sa-mRNA-LNP vaccines encoding secreted or membrane anchored HA

**Fig. 5:**
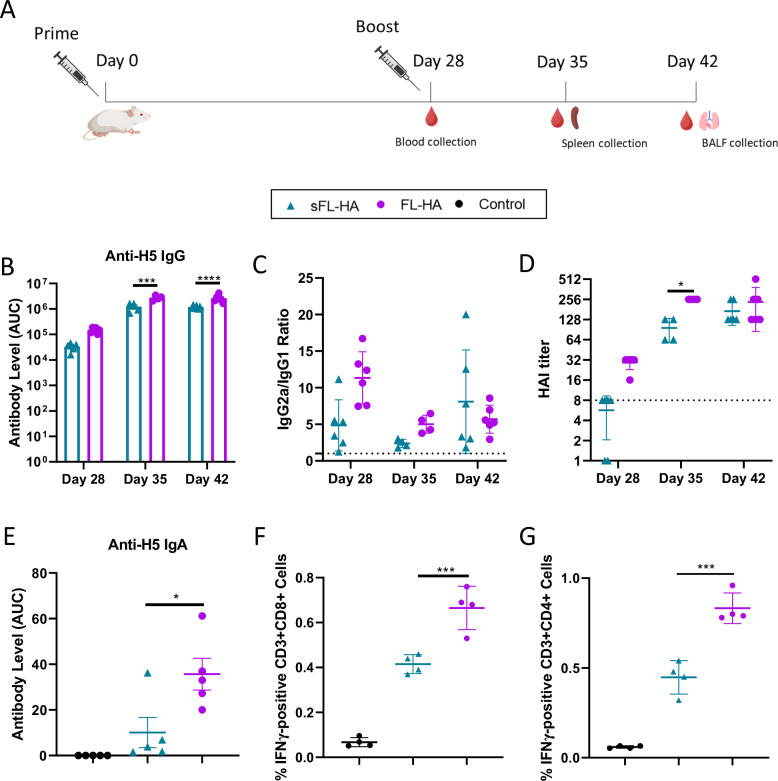
Comparison of the vaccination efficacy of sa-mRNA-LNP vaccines encoding either secreted HA or membrane anchored HA. **A** Vaccination schedule with 28-day (4-week) prime-boost interval. Mice (*n* = 10) were intramuscularly vaccinated with 1 µg sa-mRNA-LNP vaccines encoding sFL-HA or FL-HA. Mice injected with sa-mRNA-LNPs encoding luciferase served as control. **B** Serum anti-H5 IgG antibody levels against HA measured by ELISA 4 weeks after the prime (day 28 *n* = 6), one week (day 35 *n* = 4) and two weeks (day 42 *n* = 6) after the boost and shown as area under the ELISA absorbance curve (AUC). **C** The IgG2a/IgG1 ratios calculated by dividing the AUC of IgG2a by the AUC of IgG1. Dashed line indicates a value of 1. **D** HAI titers in serum samples taken four weeks after the prime (day 28), and one (day 35) and two weeks (day 42) after the boost. The detection limit is 8 and indicated as dashed line. Value sets at 1 indicate undetectable titer. **E** Anti-H5 IgA antibodies in BALFs collected two weeks after the boost. **F** and **G** depict the elicited cellular immune responses. Spleens were collected one week after the boost and splenocytes were isolated and stimulated with a HA peptide pool from A/Indonesia/CDC835/2006(H5N1). The percentage of IFN-γ positive CD3^+^CD8^+^ T cells (**F**) and CD3^+^CD4^+^ T cells (**G**) in the splenocytes were measured by flow cytometry. The significance levels in panels **E**–**G** (*p < 0.05; **p < 0.01; ***p < 0.001; ****p < 0.0001) were calculated by two-way ANOVA (**B** and **D**) or one-way ANOVA with Tukey’s multiple comparisons test. Data are represented as geometric means (**B**, **D** and **E**) or arithmetic means, Error bars represent the standard deviation. Figure **A** was created with BioRender.com.

It has been reported that genetic vaccines encoding membrane-anchored HA elicit stronger immune responses than genetic vaccines encoding secreted HA^28^. Therefore, we synthesized a sa-mRNA encoding a full-length membrane-anchored HA (FL-HA, Fig. 1) and compared its efficacy with the sa-mRNA encoding secreted full-length HA (sFL-HA). To that end, mice (*n* = 10) were intramuscularly immunized with 1 µg of LNP formulated sa-mRNA vaccines using a prime-boost schedule (Fig. 5A). In this experiment, the interval between prime and boost was 4 weeks instead of 3 weeks, as we also aimed to investigate whether a longer interval can enhance immune responses. Mice vaccinated with membrane-anchored FL-HA exhibited significantly higher anti-H5 IgG (Fig. 5B), IgG1 (Supplementary Fig. S4A) and IgG2a (Supplementary Fig. S4B) levels than mice that received the sa-mRNA encoding the secreted sFL-HA antigen (day 28 *n* = 6, day 35 *n* = 4, day 42 *n* = 6) after boost. The IgG2a/IgG1 ratios in both groups were above 1, which again indicates a Th1 response (Fig. 5C). Importantly, the FL-HA sa-mRNA group resulted, compared to the sFL-HA group, in significantly higher HAI titers on day 35 (Fig. 5D). Protective titers (HAI = 40) were almost obtained after the prime in the FL-HA group. The boost drastically increased the HAI titers in both groups. Seven days after the boost (day 35) the HAI titers were around 256 and 100 in the FL-HA and sFL-HA group, respectively. Two weeks after the boost, the HAI titers in both groups reached a similar level (Fig. 5D). On day 35, the BALFs were collected to measure the anti-H5 IgA level. The sa-mRNA encoding the membrane-anchored FL-HA also induced a significantly stronger mucosal immune response in the lungs, with anti-H5 IgA levels 3.5-fold higher than the sFL-HA group (Fig. 5E). One week after the boost, the percentages of HA specific IFN-γ secreting CD8^+^ (Fig. 5F) and CD4^+^ T cells (Fig. 5G) in spleen were also significantly higher in the FL-HA sa-mRNA vaccine group following 16 h of stimulation with the HA peptide pool. Finally, the cytokines secreted by splenocytes stimulated by HA peptide pool confirmed these data, showing a trend of higher IFN-γ and IL-2 secretion in the FL-HA sa-mRNA vaccine group (Supplementary Fig. S4E, F). Similar to the sFL-HA sa-mRNA vaccine, the FL-HA sa-mRNA vaccine did not induce IL-4 secretion by CD4^+^ and CD8^+^ T cells (Supplementary Fig. S4C, S4D, S4G). The differences between a 3-week and 4-week interval is discussed in the discussion section.

### Biodistribution of the sa-mRNA-LNP vaccine

**Fig. 6:**
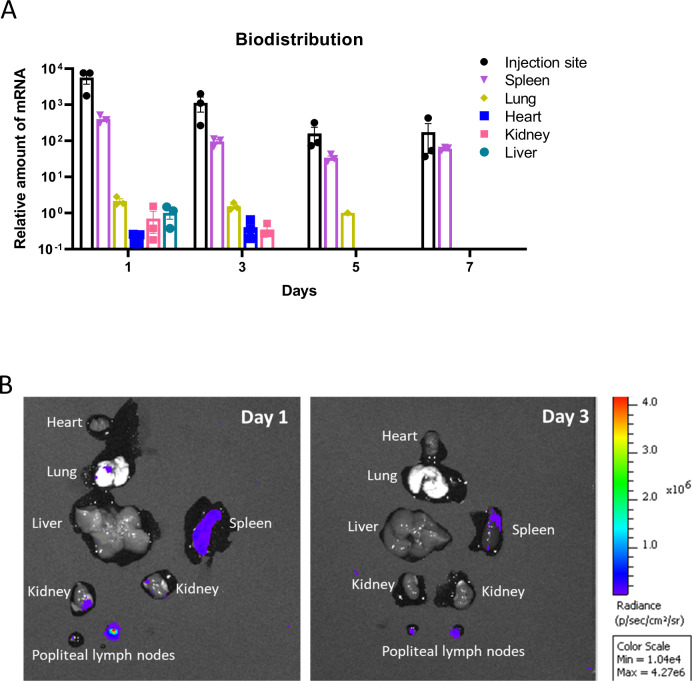
The biodistribution of the sa-mRNA-LNP vaccines in mice after intramuscular administration. **A** Mice were intramuscularly injected with 4 µg of LNP formulated sa-mRNA encoding sFL-HA. Subsequently at each time point, 3 mice were euthanized and the injection site, spleen, lung, heart, kidney, liver and brain were harvested. The presence of sFL-HA sa-mRNA in these organs was detected by RT-qPCR. The data are shown as the relative sa-mRNA level normalized to the reference gene GAPDH and the normalized sFL-HA sa-mRNA level in the liver on day 1 was set as 1. **B** Mice (*n* = 2) were intramuscularly injected with 4 µg of LNP formulated sa-mRNA encoding luciferase. The heart, lung, liver, spleen, kidney, and lymph nodes were harvested on day 1 and 3, and the bioluminescence was measured by the IVIS system.

A sa-mRNA biodistribution analysis was conducted using real-time polymerase chain reaction(RT-qPCR)^29^ after intramuscular injection of 4 µg of sa-mRNA-LNP encoding sFL-HA. Tissue samples (*n* = 3) were collected on days 1, 3, 5 and 7 after injection, from the injection site, spleen, lung, heart, kidney, liver and brain. On day 1, sFL-HA sa-mRNA was detected in all the organs except for the brain. The highest sa-mRNA levels were observed in the intramuscular injection site, followed by the spleen, the latter having approximately 100-fold higher levels than in the liver, heart, lung and kidney (Fig. 6A). From day 3 onwards, sFL-HA sa-mRNA became undetectable in the liver, and its levels in other organs decreased compared to day 1. On day 5, HA sa-mRNA levels remained detectable in the spleens and the injection sites of all mice and in the lung of 1 mouse. By day 7, HA sa-mRNA was only detectable in the spleen and the injection site. Throughout the entire period, HA mRNA was never detected in the brain.

To further confirm these findings, we intramuscularly injected mice with 4 µg sa-mRNA-LNP encoding luciferase and monitored the luciferase expression by measuring the ex vivo bioluminescent signals in the major organs and popliteal draining lymph nodes. On day 1, luciferase expression was detected in the spleen, lung, kidney and the popliteal lymph nodes (Fig. 6B). On day 3, the expression was only detected in the spleen and the lymph nodes. At the injection site, the luciferase expression was present at all time points (data not shown). In addition, we monitored the weight of mice following vaccination with 0.25 µg, 1 µg, 4 µg sFL-HA sa-mRNA-LNP or 4 µg luciferase sa-mRNA-LNP, and observed no significant differences between the vaccinated groups and the non-vaccinated PBS group. This indicates that the sa-mRNA vaccines did not induce major side effects (Supplementary Fig. S5).

## Discussion

To develop a potent influenza vaccine, both antigen-specific antibodies and T cell responses are essential for the vaccination effectiveness^30–32^. Furthermore, a local immune response in the lung is also warranted as influenza viruses predominantly enter the body via the respiratory tract and are primarily spread via respiratory droplets^33^. In this study, we engineered and investigated four sa-mRNA vaccines, of which three encoded secreted H5 HA proteins that were either in a full-length or truncated format, and a fourth encoded a full-length membrane-anchored HA. For the delivery of these sa-mRNA vaccines, we used LNPs that contained the same ionizable lipid as used in the COVID-19 BNT162b2 vaccine. As sa-mRNA is much longer than the modified mRNA in the COVID-19 BNT162b2 vaccine, we first determined the optimal N/P ratio and dose of our sa-mRNA-LNPs. Using a luciferase encoding sa-mRNA, we found that, after intramuscular administration, sa-mRNA-LNPs at an N/P ratio of 10 resulted in a higher expression than at an N/P ratios of 5 and 15 (Fig. 2A).

The first sa-mRNA vaccine encoding the H5 HA stalk domain, which was designed based on the principle of the mini HA #4900 protein vaccine^24^, generated HA-specific antibodies but failed to trigger antigen-specific CD8^+^ cellular responses (Fig. 3D). Since T cell epitopes are expected to be present in the stalk^34^, this may indicate that the folding or stability of the stalk produced by the sa-mRNA does not favor CD8^+^ T cell responses. As expected, this sa-mRNA vaccine did not induce HAI titers, as antibodies against the stalk do not interfere with the hemagglutination. Nevertheless, we cannot exclude that the elicited anti-stalk antibodies can still offer protection via e.g. antibody-dependent cellular cytotoxicity^35^.

The second sa-mRNA vaccine encoding a secreted H5 HA head domain, elicited a strong systemic response (IgG and HAI) and a highly robust T cell response (Fig. 3B–D and Supplementary Fig. S2D). This secreted H5 HA head domain is similar to the H1 head domain used in the vaccination study of Xuan et al.^36^, but it differs from the work of Khurana et al. and Chiu et al., who both used a complete HA1 sequences, which encodes besides the head domain also a part of the stalk domain^37,38^. The sa-mRNA encoding the secreted HA head domain represents a promising target to develop multivalent (sa-) mRNA vaccines, as it is less than half of the size of full-length HA protein. Indeed, Arevalo et al. evaluated a multivalent influenza vaccine by combining 20 full length HA mRNA-LNP vaccines that covered all subtypes of influenza viruses. As 2.5 µg mRNA-LNP was used for each antigen, the resulting total mRNA dose was 50 µg per mouse, which is quite high^39^. Especially with non-amplifying mRNA, the mRNA dosage could be halved when a mRNA sequence encoding the secreted HA head domain would be employed.

The third sa-mRNA vaccine encoded the secreted full-length HA (sFL-HA) and induced higher antibody levels than other secreted HA forms. The immunogenicity and efficacy of this vaccine were studied in more detail. A dose-response study demonstrated that immunization with 4 µg of the sFL-HA sa-mRNA vaccine induced significantly higher antibody responses than 1 µg or 0.25 µg (Fig. 4B, D). Nevertheless, after the boost, the 0.25 µg dose was able to elicit HAI titers that surpassed the generally accepted correlate of protection (CoP) threshold of 40, which is the minimal requirement for a vaccine to be considered effective^40^. In a previous study of Vogel et al.^14^, 0.25 µg of a sa-mRNA encoding full-length wild-type H1 HA was not able to elicit HAI titers above the protective threshold^14^. This disparity is most likely attributed to the use of a potent LNP based formulation^11^ in our work and the utilization of cellulose purified sa-mRNA vaccines, which contain less ds-RNA byproducts (Supplementary Fig. S1B) and hence improve the vaccine efficacy^41^.

T cells responses can be very valuable for influenza vaccines as cytotoxic CD8^+^ T cells can eliminate virus-infected cells. The T cell epitopes of influenza A virus, compared to its B cell epitopes, are relatively conserved, offering the potential for cross-strain protection^42^. We assessed antigen-specific T cell activation through flow cytometry and observed that a dose of 0.25 µg of sa-mRNA induced similar levels of antigen-specific CD4^+^ and CD8^+^ T cells compared to 4 µg sa-mRNA. It is noteworthy that the commercial HA peptides pool used in our study originated from a heterologous strain (A/Indonesia/CDC835/2006(H5N1)), which belongs to clade 2.1.3. The sa-mRNA vaccines in this work are based on the HA of a clade 2.3.4 HPAIV. Hence, our HPAIV sa-mRNA vaccines have the potential to induce cross-protection via T cell mediated immunity. There are two main subsets of CD4^+^ helper T cells, namely Th1 and Th2 cells, which orchestrate the immune response. Th1 cells secrete IL-2 and IFN-γ, suppressing IgG1 and IgE production, while inducing IgG2a. Conversely, Th2 cells secret IL-4 and promote IgG1 secretion^43,44^. Although IgG1 acts as the dominant isotype of virus-neutralizing antibodies, IgG2a antibodies play a crucial role in protection of mice against lethal infection by influenza H5N1 virus as well^7,45^. Our findings reveal slightly higher levels of HA-specific IgG2a than IgG1 (Figs. 4C and 5C) after primary and boost vaccination. This IgG2a isotype is highly effective in activating complement^46^ and facilitating antibody-dependent cellular cytotoxicity (ADCC)^47^. Considering the high IgG2a/IgG1 ratio (Figs. 4C and 5C) and the clear induction of HA specific T cells that produce IL-2 and IFN-γ (Fig. 4H, I and Supplementary Fig. S4E, F), we conclude that our HA sa-mRNA vaccines induced a Th1-skewed response, favoring the elimination of viral infections.

The mRNA COVID-19 BNT162b1 vaccine, encoding the secreted trimerized receptor-binding domain (RBD) of SARS-CoV-2, has demonstrated the ability to elicit robust antibody and Th1 T cell responses in humans^10^. The antibody titer and neutralizing titer obtained with this vaccine were equivalent to the mRNA COVID-19 BNT162b2 vaccine encoding a membrane-anchored full-length spike protein^6^. However, our study did not find an equal performance of the membrane-anchored and secreted full-length HA. The membrane-anchored HA outperformed the secreted HA sa-mRNA vaccines in eliciting antigen-specific immune responses, including enhanced levels of HA-specific IgG, IgG1, IgG2a, IgA, HAI titers, as well as T cell responses (Fig. 5B–G and Supplementary Fig. S4A, B). Similar to our results, Freyn et al. reported that a 1-methylpseudouridine modified non-amplifying mRNA-LNP vaccine encoding the membrane-anchored full-length H1 HA elicited a stronger antibody response compared to the secreted counterpart^28^.

In our study, the efficacy of the sFL-HA sa-mRNA-LNP vaccine was studied using both 3-week and 4-week intervals between the prime and the boost (Figs. 4 and 5). When a 3-week interval was applied, we observed that the anti-H5 IgG level did not exhibit a drastic increase 7 days after the boost (Fig. 4B), whereas the HAI titer displayed a more than 4-fold increase (Fig. 4D). Nevertheless, from 7 days to 14 days after the boost, a clear increase in IgG levels was noticed, while the HAI titer did not increase (Fig. 4B, D). Conversely, for the 4-week interval regime, a noteworthy increase in anti-H5 IgG level was observed 7 days after the boost (Fig. 5B) accompanied by a concurrent 8-fold increase in the HAI titer (Fig. 5D). However, from 7 to 14 days after the boost, neither the HAI titer nor the IgG had further increased. Overall, the 3-week and 4-week interval regimes did not generate major differences in IgG levels or HAI titers at two weeks post boost. This is in contrast with a previous report showing that a delayed-interval during vaccination with a modified non-amplifying mRNA COVID-19 vaccine enhanced humoral and T cell response^48^. A more in-depth study of the effect of longer prime-boost time intervals for sa-mRNA vaccines is needed.

To assess the biodistribution of the sa-mRNA-LNP vaccines upon intramuscular injection in mice, we used RT-qPCR to measure HA sa-mRNA levels in different organs. As expected, massive amounts of HA sa-mRNA vaccine were detected at the injection site. However, high amounts of HA sa-mRNA were also detected in the spleens, while traces of HA sa-mRNA were also present in lungs, livers and hearts. Our results are consistent with the reported biodistribution data of the BNT162b2 COVID-19 mRNA vaccine that employs a similar LNP formulation^11^. In addition, intramuscular injection of luciferase sa-mRNA-LNPs corresponded to the RT-qPCR data and demonstrated protein expression in the spleen, draining lymph node, kidney and lung. No bioluminescence was detected in the heart and liver, possibly due to the lower sensitivity compared to RT-qPCR. The detection and expression of HA sa-mRNA in the spleen and lymph nodes point to a high delivery of the sa-RNA vaccine in lymphoid tissues and immune cells, which is beneficial for vaccination applications.

In summary, our study provides comprehensive evidence that sa-mRNA vaccines coding a secreted HA head domain, a secreted full-length HA or a membrane-anchored full-length HA can induce strong humoral and cellular response, with HAI titers that are above 40, the minimal threshold for an efficient vaccine. Surprisingly, even when a low dose of 0.25 µg was used, protective HAI titers were obtained after a boost vaccination. The distribution of the sa-mRNA-LNPs to the lymph nodes and spleen most likely indicates, as confirmed by others^49,50^, that large amounts of the sa-mRNA-LNPs are internalized and expressed by immune cells, which is crucial for the efficacy of vaccines. A challenge study was not performed in this work, as mice are not sensitive to the H5N1 virus^51,52^. In sum, all the findings in this work highlight the potential of sa-mRNA-LNP vaccines as a promising approach for preventing influenza infections.

## Methods

### Cells and mice

BHK-21 cells were cultured in Dulbecco’s Modified Eagle Medium (21885108, Gibco, Thermo Fisher, USA), supplemented with 10% fetal bovine serum (S181H-500, South American origin, VWR, USA) and 100 U/mL penicillin and 100 mg/mL streptomycin (15070-063, Gibco, Thermo Fisher). Female BALB/cJRj mice (6-7 weeks old) were purchased from Janvier (France). All the in vivo experiments were strictly reviewed and approved by the Ethics Committee of the Faculty of Veterinary Medicine, Ghent University (EC2021/63).

### Synthesis and purification of self-amplifying mRNAs

The hemagglutinin gene (HA) derived from A/Anhui/1/2005(H5N1) (GenBank: HM172104) was optimized for mice expression using GenScript’s OptimumGene Codon Optimization. The corresponding DNA for secreted full-length HA, head domain HA, stalk domain HA and membrane anchored HA were synthesized by Integrated DNA technology (IDT, USA). The linear DNA sequences were designed to have overlaps with pTK161 vector, for subsequent HiFi DNA assembly (E5520S, New England Biolabs, USA). The PV01 vector contains the Venezuelan Equine Encephalitis Virus strain TC-83 5’ UTR, 3’ UTR, non-structural proteins gene and luciferase gene. After HiFi cloning, the luciferase gene in the PV01 vector was replaced by an HA gene. The self-amplifying mRNA was synthesized by in vitro transcription (IVT) as previous described^53^. Briefly, the plasmid was linearized by digested with I-SceI homing endonuclease (R0694S, New England Biolabs) for 2 h, and completeness of cleavage was confirmed by examining the template DNA through agarose gel electrophoresis. The template was purified using silica spin columns (PCR & DNA cleanup kit, T1030S, New England Biolabs). The purified templates were then used in an IVT reaction (AM1334, Thermo fisher) with co-transcriptional capping using CleanCap technology (CleanCap AU N711410, Trilink BioTechnology, USA), following the instructions provided with the kit. The mRNA was purified using an RNA cleanup kit (T2050L, New England Biolabs) and cellulose-based purification to reduce the IFN response induced by ds-RNA as described elsewhere^41^. In brief, cellulose was prewashed with chromatography buffer (10 mM HEPES [pH 7.2], 0.1 mM EDTA, 125 mM NaCl, and 16% ethanol), and the sa-mRNA was added to the prewashed cellulose and incubated for 30 min with shaking. The ssRNA was collected by centrifugation with removal of dsRNA that bind to the cellulose. The mRNA was precipitated using isopropanol, dissolved in RNase-free water and stored at −80 °C for further use.

### mRNA-LNP formulation and characterization

Sa-mRNA LNPs were formulated by rapid mixing of 3 volumes of aqueous solution containing the sa-mRNA (in sodium acetate, pH 4.5) with 1 volume of ethanolic solution (in 100% ethanol) containing the ALC-0315 lipid (HY-138170, MedChemExpress, USA), DMG-PEG2K (880151P, Avanti Polar Lipids, USA), cholesterol (700100P, Avanti Polar Lipids) and DOPE (850725 P, Avanti Polar Lipids) at a molar ratio of 50 : 1.5 : 38.5 : 10. After formulation, mRNA-LNPs were subjected to dialysis in a dialysis cassette (66003, Thermo Fisher) against DPBS (14190144, Thermo Fisher) to remove ethanol.

Sa-mRNA-LNP formulations were diluted in 20 mM HEPES buffer (pH 7.4, 15630080, Thermo Fisher) and transferred into polystyrene cuvettes to measure particle size, polydispersity and zeta potential by dynamic light scattering (Zetasizer Nano-ZS, Malvern Panalytical B.V., Belgium). The diameters are reported as Z-average values. The mRNA encapsulation efficiency was measured by Quanti-it RiboGreen assay (R11490, Thermo Fisher) according to the provided instruction manual. Fluorescence emission intensity was recorded on a Cytation 5 (Agilent, USA) set at 485 nm excitation and 525 nm emission. mRNA encapsulation in LNP samples was calculated by comparing the fluorescence intensity of the mRNA-binding fluorescent dye RiboGreen® in the absence (signal from unencapsulated mRNA) and presence (signal from both encapsulated and unencapsulated) of 2% Triton X-100 (M236-10ML-5PK, VWR) to dissolve the LNP. Two-standard curves were made by using the standard sample of the kit, one curve with Triton x-100, one without Triton X-100. The encapsulation efficiency is calculated using the following equation: encapsulation efficiency (%) = ((mRNA total − mRNA unencapsulated)/mRNA total) × 100%. The most optimal N/P ratio and dose-response curve of the sa-mRNA-LNPs were determined after intramuscular injection of LNP formulated sa-mRNAs in BALB/c. The expression kinetics of the luciferase was measured after subcutaneous injection of 200 µl D-luciferin (15 mg/ml) using in vivo optical imaging with an IVIS Spectrum In Vivo Imaging System (PerkinElmer, USA).

### Western blot

To detect the HA protein in vitro, BHK cells were transfected with the sa-mRNA vaccines using Lipofectamine MessengerMax (LMRNA003, Invitrogen, USA). The supernatant was collected 24 h after transfection and centrifuged at 400 × *g* for 5 min 4 °C. The cell supernatant was concentrated using a centrifugal filter unit (Amicon Ultra-4 Centrifugal filter unit, ufc801008, Merck, USA). Simultaneously, the cells were wash twice with PBS, and 200 µL RIPA lysis buffer containing a protease inhibitor cocktail was added to the plate on ice. The plate was then shaken for 30 min. The lysate was centrifuged at 12,000 × *g* for 5 min at 4 °C. Subsequently, 9 µl supernatant was mixed with 1 µl glycoprotein denaturing buffer (10x) (NEB, P0704S, USA) and heated up to 100 °C for 10 min. Next, 2 µl GlycoBuffer 2 (10X), 2 µl 10% NP-40, 6 µl distilled H2O and 1 µl PNGase F (all from NEB, P0704S, USA) were added to the denatured samples and incubate at 37 °C for 1 h Finally, 4 µl Laemmli SDS sample buffer (6x) (J61337.AC, Thermo fisher) was added. Subsequently, 10 µL of the samples were loaded onto a gel (Bio-Rad 4569033) for electrophoresis. After electrophoresis, the proteins were transferred to a PVDF membrane that was subsequently blocked with 5% skim milk and incubated with HRP-conjugated anti-Flag antibody (ab49763, Abcam, USA) or anti-HA antibody (MA5-30009, Thermo fisher, USA) for 2 h. Next, the membrane incubated with anti-HA antibody was incubated with HRP-conjugated goat anti-mouse IgG(H + L) (31430, Invitrogen) for 1 h. The protein bands were visualized by adding an ECL western blotting detection kit and imaged using the ChemiDoc XRS+ system (Bio-rad).

### Vaccination experiments in mice

All injections of mice were administered intramuscularly into the quadriceps muscle (50 µl), and sa-mRNA encoding luciferase was used as a control. A prime-boost regime was employed for the vaccination studies with a 3 or 4-week interval.

To investigate the three sa-mRNA encoding secreted HAs (sFL-HA, sHD-HA, sSD-HA), mice (*n* = 3) (n indicates the number of mice per group) were injected intramuscularly at day 0 with 1 µg sa-mRNA-LNP using a prime-boost schedule with a 3-week interval. Sa-mRNA encoding luciferase was used as control. The blood samples were collected from the tail vein on day 21 and day 33. Mice were euthanized by cervical dislocation and spleens were harvest for further analysis on day 33.

To compare the immune response at different administered dose, four groups, each consisting of 12 mice, were intramuscularly injected with 0.25 µg, 1 µg or 4 µg sFL-HA or 4 µg of control sa-mRNA LNP, respectively, on day 0 and day 21. Blood samples were collected from random mice’s tail veins on day 21 (*n* = 9), 28 (*n* = 4) and 35 (*n* = 6). On day 28, spleens were collected from 6 mice in each group. On day 35, mice were euthanized by cervical dislocation, and BALF samples were collected. The BALF was collected following the method described by Lien^54^. Briefly, 1 ml sterile PBS was gently injected into the murine lung and then gently aspirated. The collected fluid was centrifuged at 400 × *g* for 5 min at 4 °C. The fluid was stored at −80 °C until further use.

In the comparison experiment between sFL-HA and FL-HA, 10 mice in each group were intramuscularly administered with 1 µg sFL-HA, FL-HA or the control sa-mRNA LNP on day 0 and day 28. Blood samples were taken from random mice’s tail vein on day 28 (*n* = 6), day 35 (*n* = 4), and 42 (*n* = 6). On day 35, spleens were collected from 4 mice in each group. On day 42, BALF samples were collected after euthanasia.

### ELISA

HA-specific antibody levels were determined using ELISA. Nunc MaxiSorp plates (44-2404-21, Thermo fisher) were coated with 25 ng A/Anhui/1/2005 Hemagglutinin HA Protein (11048-V08B, Sino Biological, China) overnight at 4 °C. The plate was washed three times with washing buffer (PBS with 0.05% tween-20) and then blocked with assay buffer (DS98200, Thermo fisher) for 1 h. After washing, serially diluted mouse serums were added to the plate and incubated at room temperature for 2 h. The plate was washed and the secondary HRP-conjugated goat anti mouse IgG (H + L) (31430, Invitrogen), IgG1(PA1-74421, Thermo Fisher) or IgG2a (A-10685, Thermo Fisher) antibody was added and incubated at room temperature for 45 min followed by 4-5 washes. TMB solution was then added to the plate and incubated for 15 min. The reaction was stopped by adding 100 μL of stop solution, and the absorbance at 450 nm was measured using a Cytation 5. The antibody levels were determined as the area under curves (AUC) of HA-specific antibody ELISA curves. The Cytokines from supernatant of stimulated splenocytes (as described in the Flow cytometry section) were measured using the corresponding ELISA kit, IFN-γ (88-7314, Invitrogen), IL-2 (88-7024, Invitrogen), IL-4 (88-7044, Invitrogen), IL-10 (88-7105, Invitrogen), following the provided instructions.

### Flow cytometry

Seven or twelve days after boost immunization, spleens were collected from mice. The spleens were homogenized using a syringe plunger, and the red blood cells were lysed using ACK lysing buffer (A1049201, Thermo Fisher). A total of 10^6^ splenocytes were stimulated with PepMix™ Influenza A (HA/Indonesia (H5N1) (PM-INFA-HAIndo, Jpt, Germany) or with cell stimulation cocktail as a positive control (00-4970, Invitrogen) for 6 or 16 h. Cytokine secretion was inhibited by a mixture of Monensin Solution (00-4505-51, Thermo Fisher) and eBioscience™ Brefeldin A Solution (00-4506-51, Thermo Fisher). After stimulation, the Fc receptors were blocked with Rat anti-mouse CD16/CD32 (553142, BD Biosciences, USA). Splenocytes were then incubated with Alexa Fluor® 488 anti-mouse CD3 antibody (100210, Biolegend, USA), PerCP/Cyanine5.5 anti-mouse CD4 Antibody (116011, Biolegend), Alexa Fluor® 700 anti-mouse CD8a Antibody (100729, Biolegend) and eBioscience Fixable Viability dye eFluor 506 (65-0866-14, Invitrogen) for 15 min. Following permeabilization, splenocytes were stained with PE anti-mouse IFN-γ Antibody (163503, Biolegend) and APC anti-mouse IL-4 Antibody (504105, Biolegend). The fluorophores were detected using a CytoFLEX flow cytometer (Beckman Coulter, USA). Gating strategy is shown in Supplementary Fig. S6.

### HAI assay

To determine the levels of protective anti-HA antibodies in the collected serum samples, an HAI assay was performed as described^55^. First, HA titration of the H5N1 virus particle (07/290, NIBSC, UK) was determined using a 0.75% turkey blood cell suspension, to quantify the virus particles needed for the hemagglutination inhibitor assay. Next 10 µL of mouse serum was mixed with 30 µL cholera filtrate (c8772, sigma) and incubated at 37 °C overnight, followed by incubation at 56 °C for 30 min to inactivate cholera filtrate. Each well of a 96 V-bottom plate was filled with 25 µL PBS. Subsequently the first wells of each row received 25 µL of serum mixture and 2-fold serial dilutions were performed. In the last wells 25 µl was discarded. The lowest dilution factor of serum is 8. Subsequently, 25 µL of the inactivated strain matched H5N1 virus containing 4 HA units was added to each well. The plate was gently tapped and incubated for 30 min at room temperature. Finally, 50 µL of 0.75% turkey blood cell suspension was added to each well and incubate for 30 min. The plate was then tilted vertically for 25 s before read out. After obtaining the results, HI titers were loaded into GraphPad Prims (version 10.1.10), and the “geometric mean with geometric SD” was selected for the analysis.

### mRNA biodistribution analysis

BALB/c mice were intramuscularly injected with either 4 µg of sFL-HA sa-mRNA-LNP vaccine or PBS. In the HA sa-mRNA-LNP vaccine group, 3 mice were euthanized on day 1, 3, 5, and 7 post injection. Tissues including the injection site muscle, spleen, heart, liver, kidney, lung and brain were harvested, and less than 100 mg tissue was homogenized. Total mRNA was extracted using the Trizol chloroform method, and the total RNA concentration was measured using a nanodrop. For reverse transcription, 1 µg RNA was subjected to the ImProm-II™ Reverse Transcription System (A3800, Promega). After the transcription, the reaction was supplemented with 180 µl PCR-grade H_2_O, resulting in a final volume of 200 µL. The qPCR reaction consisted of 5 µL of KAPA SYBR® FAST qPCR master mix (2x), 0.5 µL of 10 µM forward/reverse primer, 2 µL of cDNA template, and 2 µL of PCR-grade H_2_O. The q-PCR procedure involved an initial step at 95 °C for 3 min, followed by 40 cycles of denaturation at 95 °C for 3 seconds and annealing/extension at 60 °C for 20 s. The reaction was then held at 25 °C. The GAPDH gene acted as a reference gene and was amplified using following primers: Forward: TGGAAAGCTGTGGCGTGAT, Reverse: ACACATTGGGGGTAGGAACAC. The HA in the sFL-HA sa-mRNA was amplified using the following primers Forward: CCAACCAGGAGGATCTTCTTATC, Reverse: TTGCTCTTCGTGGCAATCT. The relative sa-mRNA level was calculated by subtracting the HA cycle threshold (CT) value from GAPDH CT value, then calculating the average ΔCt value of the liver on day 1, and finally calculating 2 to the power of ΔΔCt (ΔCt _liver_ -ΔCt) representing the relative mRNA expression level.

For the ex vivo imaging, sa-mRNA encoding luciferase was injected into mice, and on day 1 and 3, mice were euthanized after first injecting them subcutaneously with 200 µl D-luciferin (15 mg/ml). The heart, lung, spleen, liver, kidney and draining lymph node were collected and the bioluminescence was measured in an IVIS Spectrum In Vivo Imaging System (PerkinElmer).

### Dot blot analysis of dsRNA contamination

500 ng of mRNA in 5 µl RNase free water were loaded in duplicate onto a positively charged nylon membrane (Whatman Nytran SuPerCharge, 10416230, Sigma-Aldrich). After drying, the membrane was blocked with 5% (w/v) non-fat dried milk in TBS-T buffer (0.1% (v/v) Tween-20 in TBS) for 1 h at room temperature. After three washes with TBS-T buffer, the membrane was incubated at room temperature for 2 h with mouse J2 anti-dsRNA murine antibody (76651 L, Cell Signaling Technology) diluted 1:2000 in TBS-T buffer containing 1% (w/v) non-fat dried milk. Next, the membranes were washed three times with TBS-T buffer and incubated for 1 h at room temperature with horseradish peroxidase (HRP)-conjugated goat anti-mouse immunoglobulin G (IgG) (31430, Invitrogen) diluted 1:10,000 in TBS-T buffer containing 1% (w/v) non-fat dried milk. After washing the membranes four times with TBS-T buffer, detection of the target dsRNA on the membrane was performed using tetramethylbenzidine (TMB) solution (002023, Thermo Fisher).

### Supplementary information

Supplemental Information

## Data Availability

All data supporting the findings of this study are included in this manuscript or supplementary data. All relevant data are available from the corresponding authors upon reasonable request.
