# Supplementary material for: Immunogenicity and biodistribution of lipid nanoparticle formulated self-amplifying mRNA vaccines against H5 avian influenza
Source: NPJ Vaccines. 2024 Aug 3;9:138. doi: 10.1038/s41541-024-00932-x (PMC11298010; doi:10.1038/s41541-024-00932-x)
Supplement: Supplementary file 1 — Supplemental Information [file 41541_2024_932_MOESM1_ESM.pdf]

Supplementary Figure 1

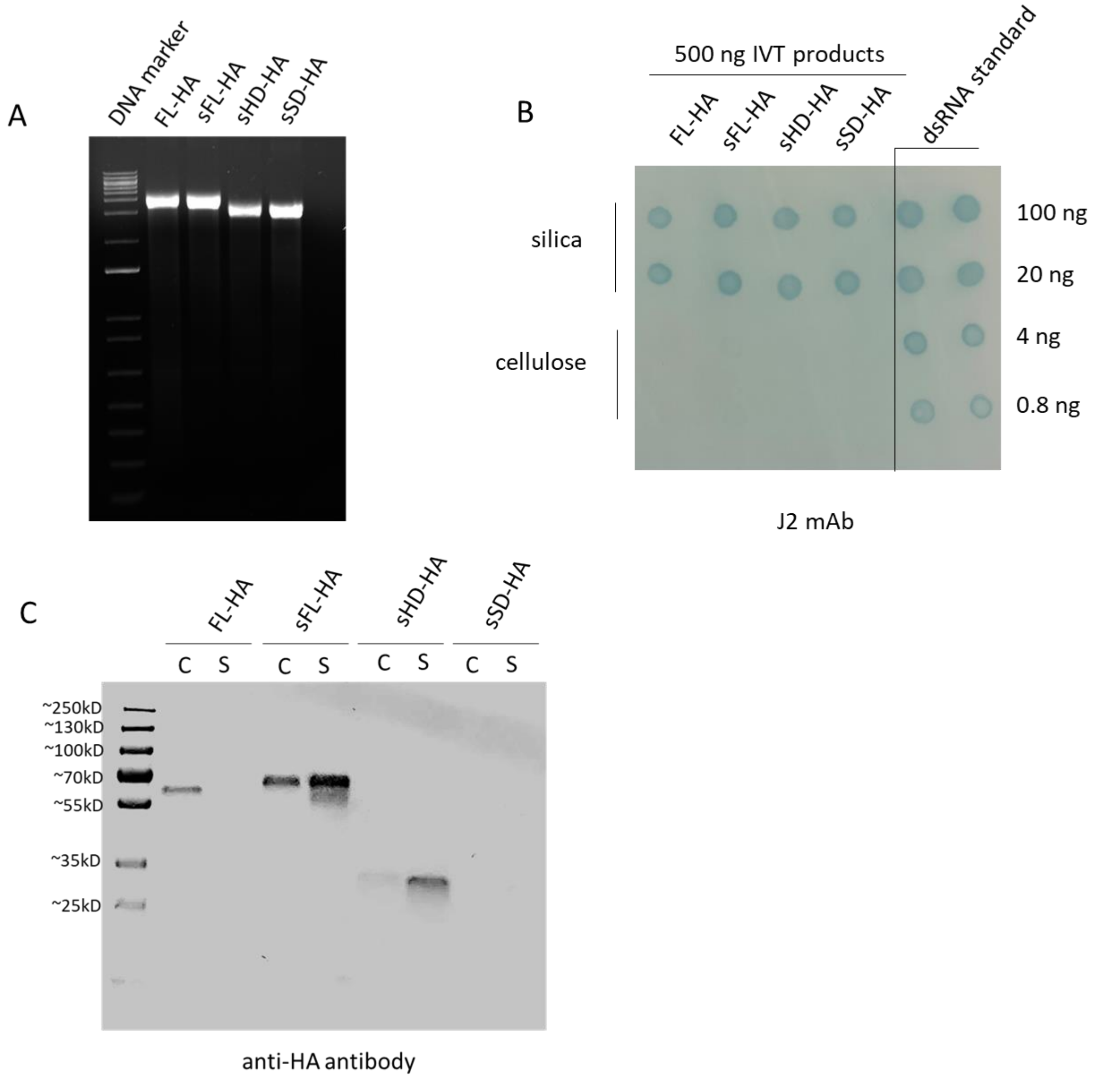

Supplementary Figure 1. Agarose gel electrophoresis of the sa-mRNA vaccines and dot blot showing dsRNA contamination in sa-mRNA vaccines purified by silica or cellulose and western blot showing the protein molecular weight ladder.

(A) After cellulose purification, 1 µg sa-mRNA from vaccine was loaded on an agarose gel. A DNA marker was used to estimate the size of the sa-mRNAs. (B) Dot blot visualizing the amount of dsRNA found in 500 ng sa-mRNA vaccine purified by silica (run in duplicate, first 2 rows) or silica followed by cellulose (run in duplicate 3rd and 4th row). DsRNAs were detected using the J2 dsRNA monoclonal antibody followed by incubation with HRP conjugated goat-anti mouse antibody. dsRNA standards ranging from 100 ng to 0.8 ng were loaded in duplicate on the right 2 column. Dot blot was visualized

using tetramethylbenzidine (TMB) solution. (C) BHK cells were transfected with FL-HA, sFL-HA, sHD-HA and sSD-HA sa-mRNA. Cell lysis (C) or supernatant (S) collected 24 hours later were loaded on gel. After transferring protein to PVDF membrane, monoclonal antibody against HA (Thermofisher, Cat. MA5-30009) and goat-anti mouse antibody (Invitrogen, Cat. 31430) were used to detect the HA proteins.

Supplementary Figure 2

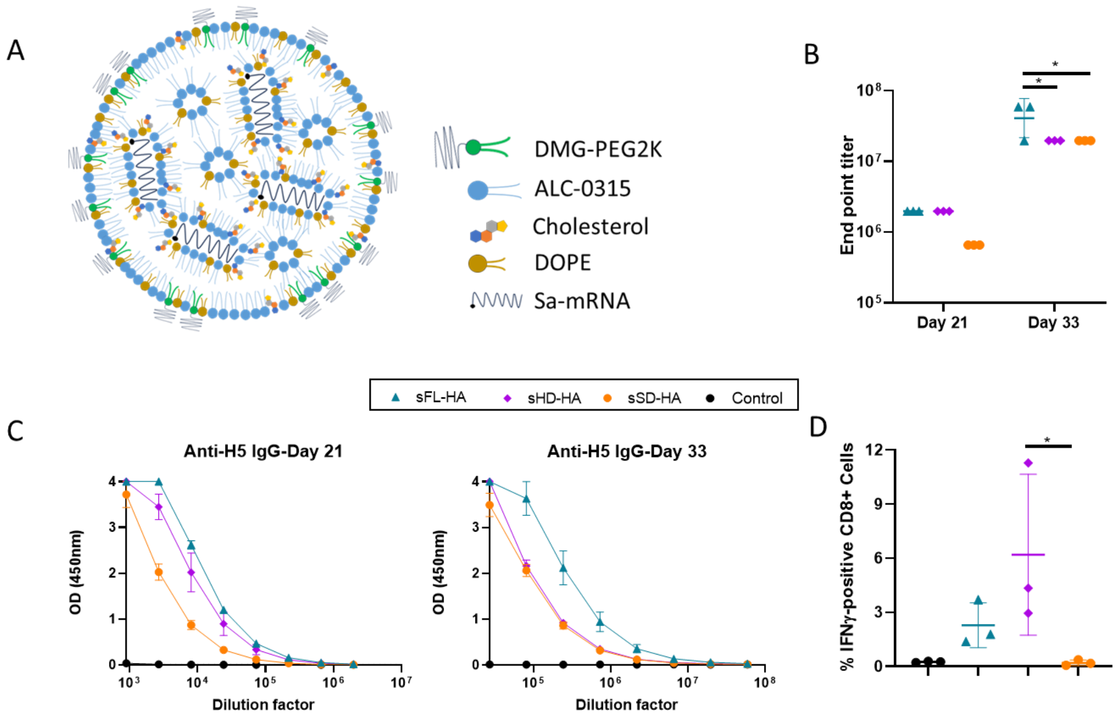

Supplementary Figure 2. Immune responses after vaccination of mice with sa-mRNA-LNPs encoding secreted HA antigens.

*Mice were intramuscularly vaccinated with 1 µg LNP formulated sa-mRNA encoding secreted HA antigens using a prime-boost schedule with a three week interval. Control mice received 1 µg of LNP formulated sa-mRNA encoding luciferase. Blood samples were collected three weeks after the prime (day 21) and twelve days after the boost (day 33). Spleens were collected 12 days after the boost (day 33). (A) Schematic structure of the sa-mRNA-LNPs used in this study, (B) total anti-H5 IgG antibody levels shown as end point titers and (C) as absorbances as a function of serum dilution. (D) The percentage of IFN-γ positive CD8+ T cells in splenocytes collected 12 days after the boost. The splenocytes were stimulated for a longer time, i.e. 48h instead of 6h, with a pool of peptides derived from HA (A/Indonesia/CDC835/2006(H5N1)). The significance level (n = 3; p<0.05, *) was calculated using two way ANOVA (B) or one-way ANOVA (D) with Tukey's multiple comparisons test. Geometric means (B) or means (n = 3) are shown and the error bars represent the standard deviation (SD).*

Supplementary Figure 3

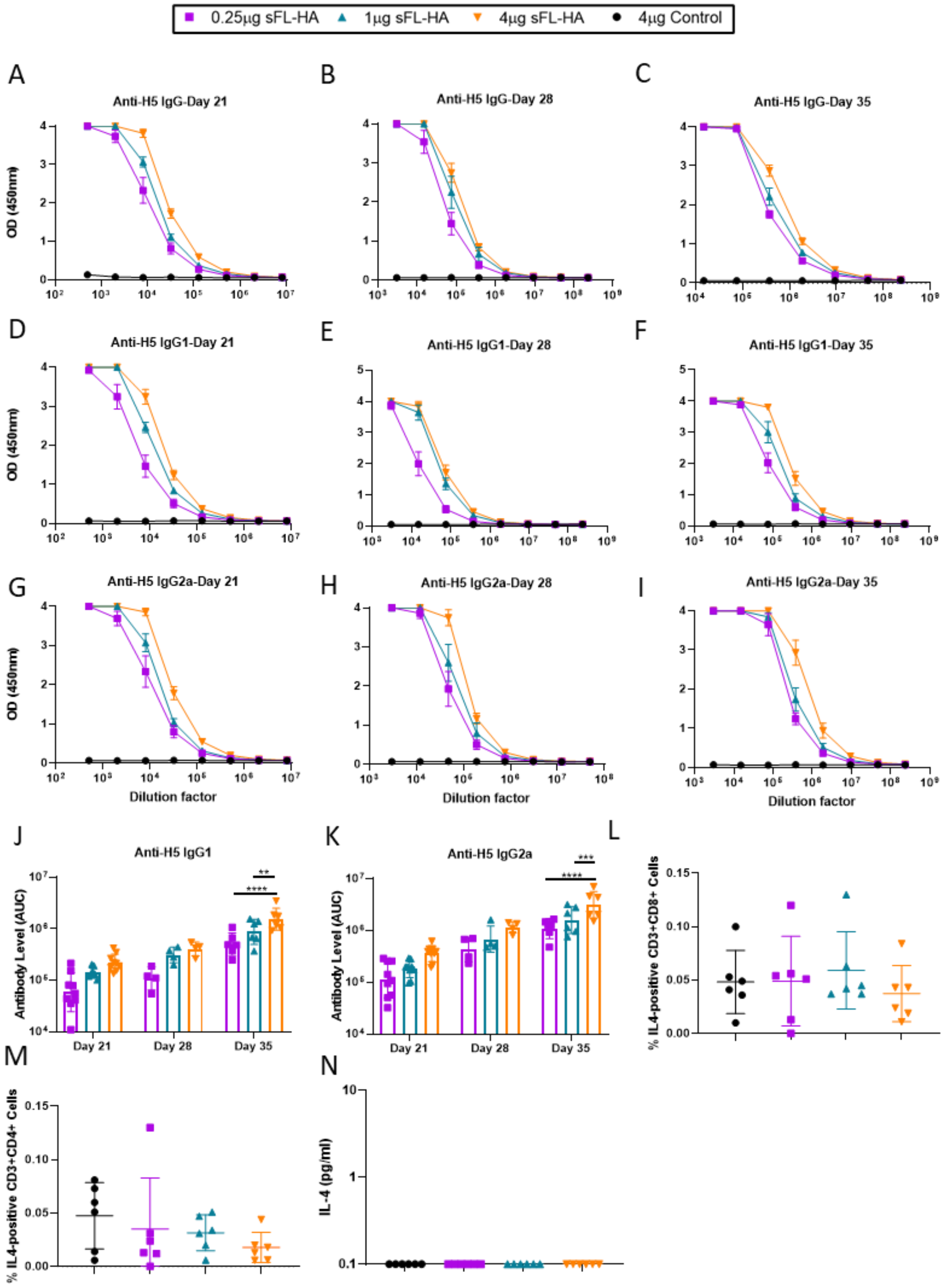

Supplementary Figure 3. Total serum IgG, IgG1 and IgG2a titers and cellular immune responses after vaccination with different doses of sa-mRNA encoding secreted full-length HA (sFL-HA).

Mice were intramuscularly vaccinated with 0.25, 1 or 4µg of LNP formulated sa-mRNA encoding sFL-HA using a prime-boost schedule with a three week interval. Control mice received 4 µg of LNP formulated sa-mRNA encoding luciferase. Serum samples (day 21 n = 9, day 28 n = 4, day 35 n = 6) were collected three weeks after the prime (day 21, panels A, D, G), one week after the boost (day 28, panels B, E, H) and two weeks after the boost (day 35, panels C,F,I) to measure HA-specific IgG (A-C), IgG1 (D-F), and IgG2a (G-I) levels. The antibody levels are shown as absorbance values as a function of the serum dilution factor. Panels J and K show the area under the curves of respectively graphs D, E, F and G, H, I. Splenocytes were collected one week *after the boost and the percentage of IL-4 producing CD3+CD8+ T cells (L) and CD3+CD4+ T cells (M) after stimulation with a pool of peptides derived from HA (A/Indonesia/CDC835/2006(H5N1)) was determined. Panel (N) shows the amount of IL-4 secreted in the supernatant of stimulated splenocytes. The significance level (p< 0.01, **; p< 0.001, ***; p<0.0001, ****) was calculated using two way ANOVA (J and K) or one-way ANOVA (L-N) with Tukey's multiple comparisons test. Data are represented as geometric means (J and K) or means and the error bars represent the relative SD.*

Supplementary Figure 4

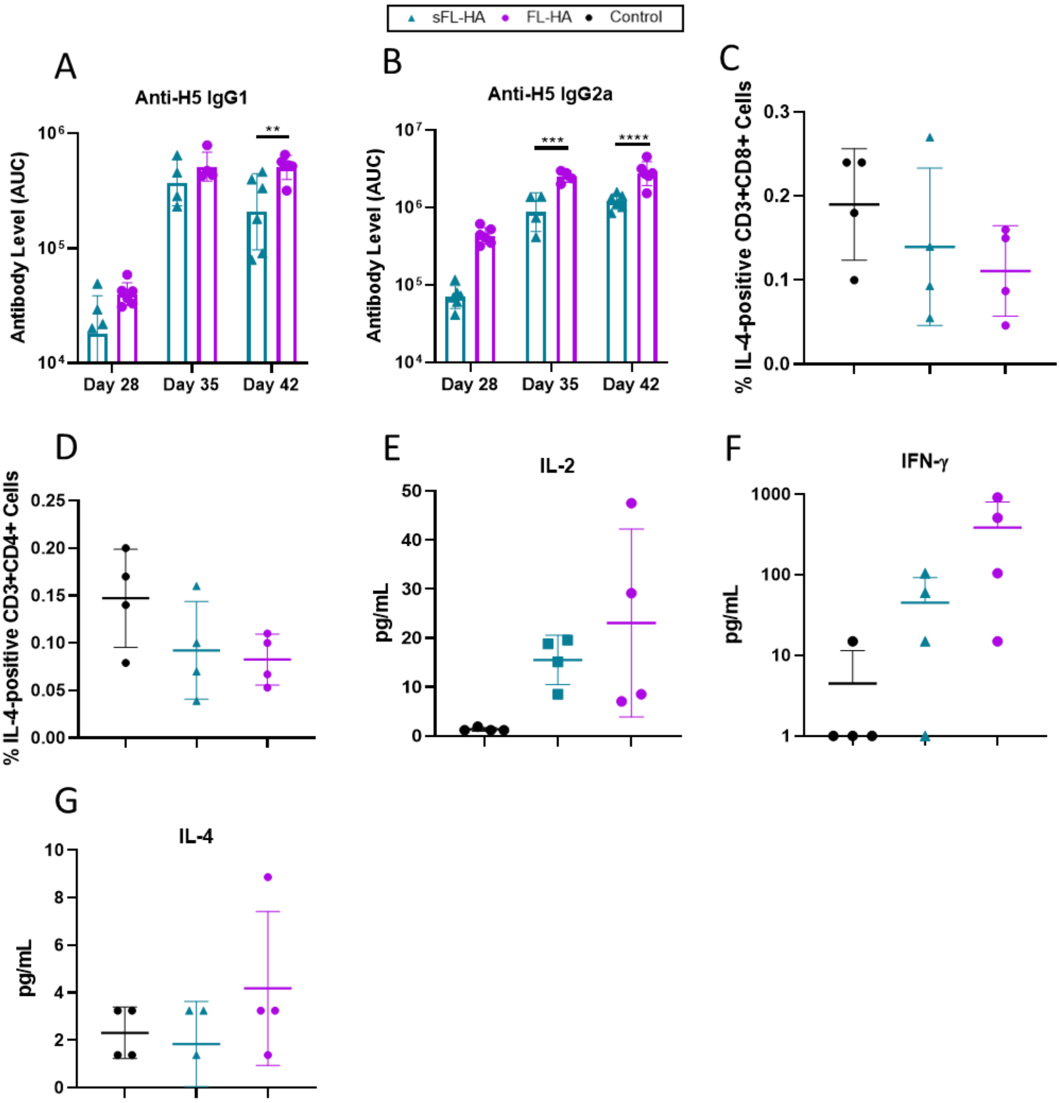

Supplementary Figure 4. Serum titers (IgG1, IgG2a) and cellular immune responses after vaccination with LNP formulated sa-mRNAs encoding secreted full-length (sFL) or membrane-anchored full-length (FL) HA.

Mice were intramuscularly vaccinated with 1µg LNP formulated sa-mRNA encoding sFL-HA or FL-HA using a prime-boost schedule with a 4 week interval. Control mice received 1 µg LNP formulated saRNA encoding luciferase. Serum samples (day 28, n = 6; day 35, n = 4; day 42, n = 6) were collected four weeks after the prime (day 28), one and two weeks after the boost (day 35 and 42) to measure HA-specific IgG1 (A) and IgG2a (B) serum levels. No HA-specific antibodies could be detected in the serum samples of control mice. The antibody levels are shown as area under the curve.

*Splenocytes were collected one week after the boost and the percentage of IL-4 producing CD3+CD8+ T cells (C) and CD3+CD4+ T cells (D) after stimulation with a pool of peptides derived from HA (A/Indonesia/CDC835/2006(H5N1))) was determined. The amount of IL-2 (E), IFN-γ (F) and IL-4 (G) secreted in the supernatant by stimulated splenocytes. Figure A and B are shown as geometric mean and SD. Figure C-G are shown as mean and SD. The significance levels (p<0.05, *; p< 0.01, **; p<0.001, ***; p<0.0001, ****)) were calculated by two-way ANOVA(A and B) or one-way ANOVA (C-G) with Tukey's multiple comparisons test.*

Supplementary Figure 5

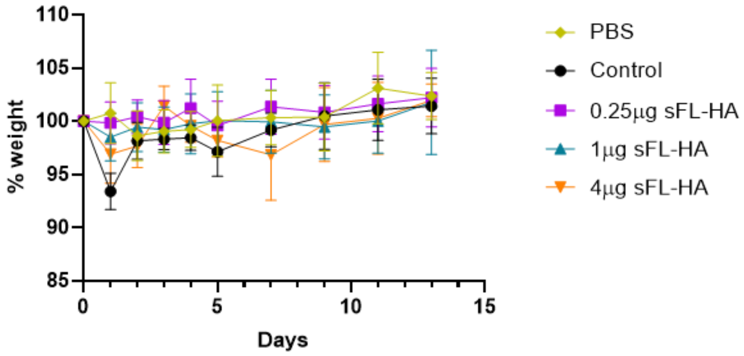

Supplementary Figure 5. Percentage change in body weight following administration of different doses of LNP formulated sa-mRNA encoding secreted full-length HA (sFL-HA) .

Mice (n = 6) were intramuscularly injected on day 0 with 0.25 µg, 1 µg or 4 µg of LNP formulated sa-mRNA encoding sFL-HA. Mice (n = 6) were intramuscularly injected with either 4 µg of LNP formulated sa-mRNA encoding luciferase (control) or PBS only (n = 4). The body weight of the mice was monitored for 13 days, with the initial weight on day 0 being set as 100%.

Supplementary Figure 6

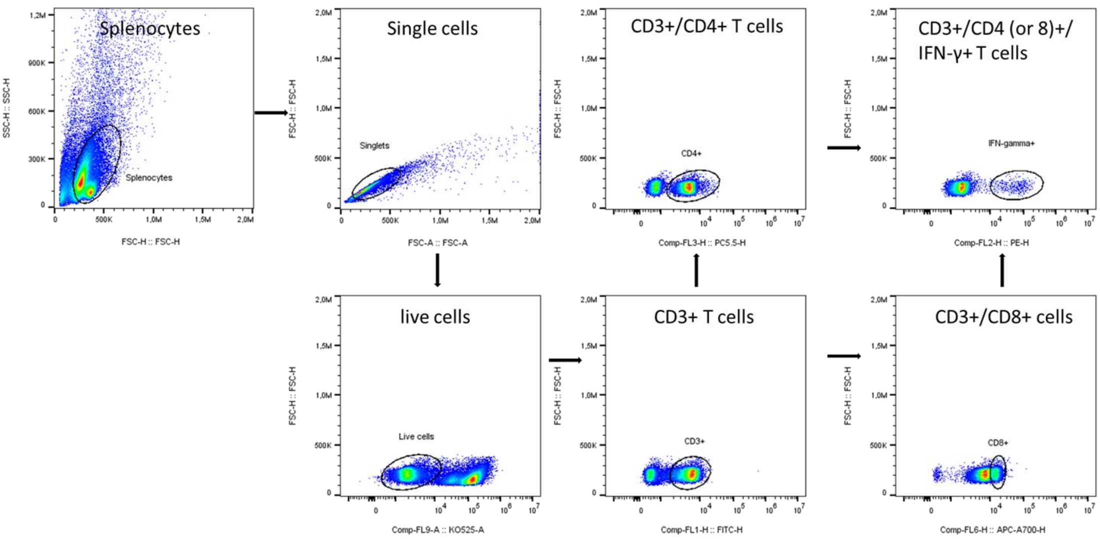

Supplementary Figure 6. Illustration of the gating strategy to identify CD4+ IFN-γ+ or CD8+ IFN-γ+ T cells within the splenocytes

Supplementary Figure 7

A

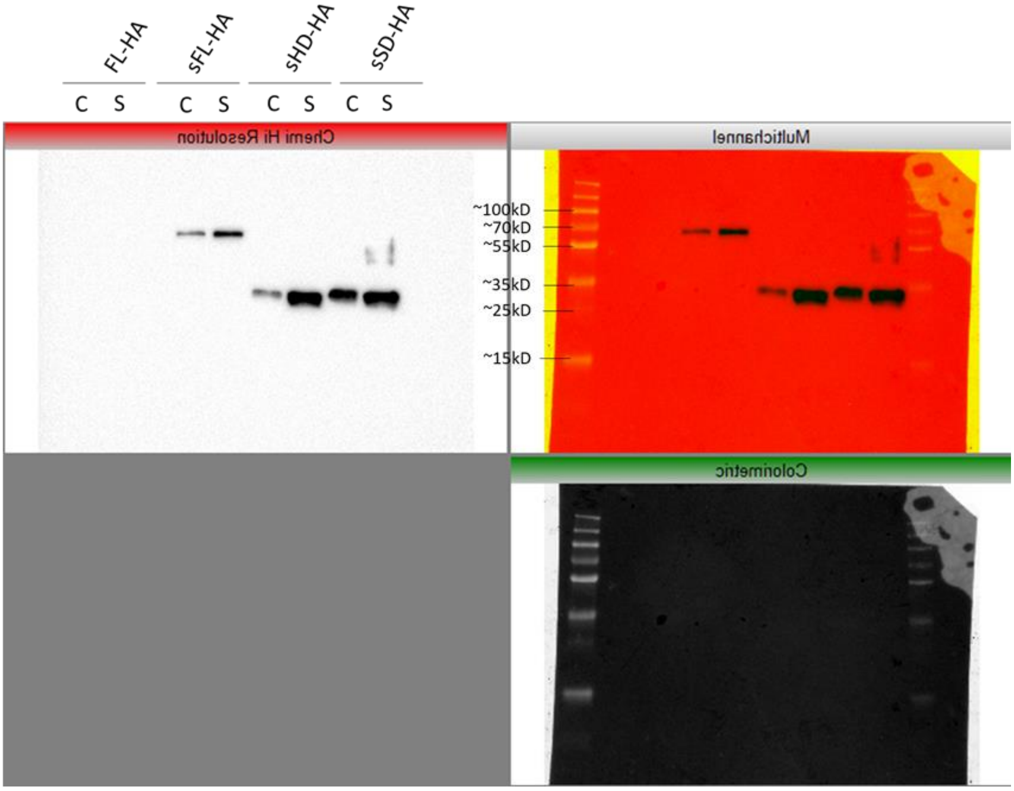

B

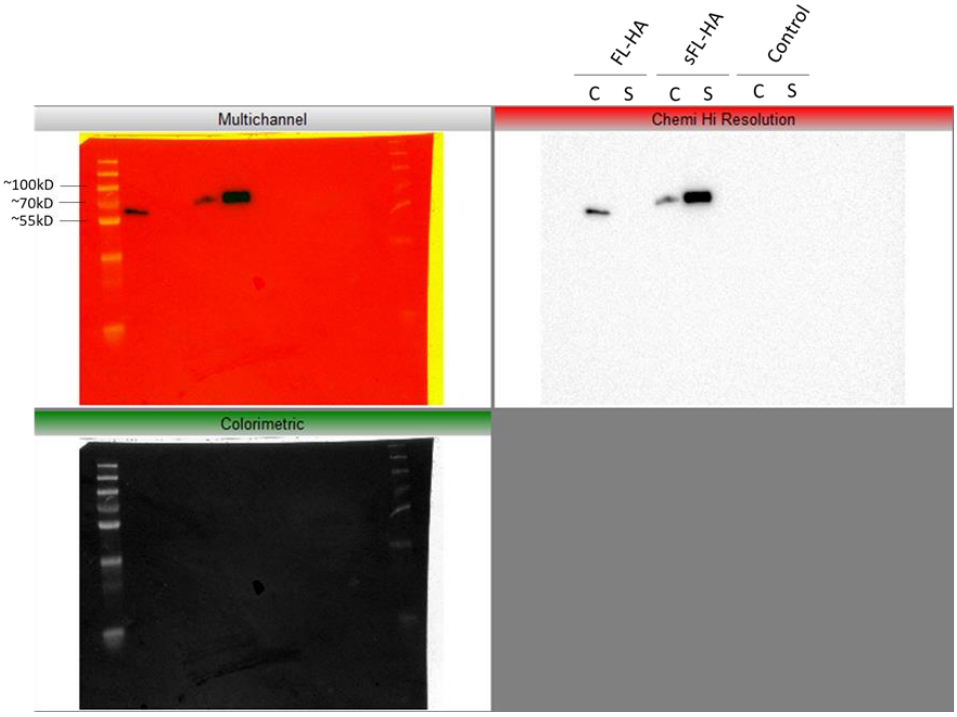

Supplementary Figure 7. Uncropped and unprocessed images used to generate Fig. 1C (Fig.S7A) and 1D (Fig.S7B). The pictures are imaged using the ChemiDoc XRS+ system (Bio-rad) and image lab software (Bio-rad).

Supplementary Table 1. The physiochemical parameters of the sa-mRNA-LNPs used in the different vaccination experiments.

Figure 3					
		Control	sFL-HA	sHD-HA	sSD-HA
Prime	size (nm)	111,5	142,7	107,1	102
	zetapotential (mV)	-3,66	-2,54	-3,17	-2,38
	encapsulation efficiency (%)	98,3	97,6	92,6	97,2
Boost	size (nm)	108	118,5	141,6	149,3
	zetapotential (mV)	-2,52	-2,55	-1,83	-2,26
	encapsulation efficiency (%)	92,1	93,5	94,6	92,4
Figure 4					
		Control	sFL-HA		
Prime	size (nm)	122,2	108,3		
	zetapotential (mV)	-1,85	-0,725		
	encapsulation efficiency (%)	93,1	94,4		
Boost	size (nm)	112	126,1		
	zetapotential (mV)	-3,78	-1,09		
	encapsulation efficiency (%)	95,6	92,2		
Figure 5					
		Control	sFL-HA	FL-HA	
Prime	size (nm)	116,3	135,6	145	
	zetapotential (mV)	3,5	1,24	1,04	
	encapsulation efficiency (%)	94,8	96,4	92,1	
Boost	size (nm)	113,2	121,7	109,9	
	zetapotential (mV)	1,85	3,22	2,41	
	encapsulation efficiency (%)	97,4	94,3	96,6	
